# The Dissociation between Adult Intelligence and Personality with Respect to Maltreatment Episodes and Externalizing Behaviors Occurring in Childhood

**DOI:** 10.3390/jintelligence6030031

**Published:** 2018-07-09

**Authors:** Carmen Flores-Mendoza, Sergio Escorial, Oscar Herrero, Roberto Colom

**Affiliations:** 1 Department of Psychology—Laboratory of the Individual Differences Assessment, Universidade Federal de Minas Gerais, Av. Antonio Carlos, 6627-Pampulha, Belo Horizonte 31270-901, Minas Gerais, Brazil; 2 Department of Methodology, Faculty of Psychology, Universidad Complutense, Av. Séneca, 2, 28040 Madrid, Spain; sergio.escorial@psi.ucm.es; 3 Penintentiary Center of Cáceres, Arroyo Valhondo, 1, 10004 Cáceres, Spain; psicoski@gmail.com; 4 Department of Biological Psychology and Health, Universidad Autónoma de Madrid, Ciudad Universitaria de Cantoblanco, 28049 Madrid, Spain; roberto.colom@uam.es

**Keywords:** maltreatment, externalizing behavior, intelligence, personality, childhood, adulthood

## Abstract

Here we analyze the simultaneous relationships among five variables. Two refer to childhood (episodes of various forms of maltreatment and externalizing behaviors), whereas three refer to early adulthood (intelligence, personality, and socialization difficulties). The 120 individuals considered for the present report were invited from the 650 schoolchildren participating in the Longitudinal Study of Intelligence and Personality (Minas Gerais, Brazil). The complete sample was recruited in 2002 (T1; mean age = 10.0; standard deviation (SD) = 2.2) and 120 were tested again in 2014-17 (T2; mean age = 23.5; SD = 2.2). Externalizing behaviors were registered at T1, whereas the remaining variables were obtained at T2. These were the main results: (1) externalizing behaviors predict future social effectiveness (as estimated by the general factor of personality derived from the NEO Personality Inventory-Revised (NEO-PI-R) and socialization difficulties computed from the socialization scale (SOC)) and future intelligence performance (as assessed by a set of fluid and crystallized tests); (2) episodes of self-reported childhood maltreatment predict social effectiveness, but not intelligence; (3) maltreatment and externalizing behaviors are unrelated; and (4) social effectiveness (personality) and intelligence are unrelated. Therefore, the findings support the dissociation between adult intelligence and personality with respect to maltreatment episodes and externalizing behaviors occurring in childhood. Implications of these findings for social policies aimed at preventing adult socially ineffective personalities are underscored.

## 1. Introduction

### 1.1. The Externalizing Spectrum

The effects of childhood experiences in adult behavior have been a classic topic in psychology since the early years of psychoanalysis. Despite the fact that cognitive and behavioral models have devoted less attention to childhood, current psychological explanations of abnormal behavior often rely on concepts such as dysfunctional attachment bonds, under the assumption that they extend their influence to the adult years.

An early source of negative adult outcomes is the presence of childhood disturbing behaviors. Problem behaviors such as impulsivity, aggression, violence, antisocial behavior and substance misuse have been summarized by the label “externalizing spectrum” [1]. An estimated 5% of the population (mostly men) displays these problems with noteworthy severity [2]. They seem to have developmental stability and persist from childhood to adolescence and adulthood [3,4,5]. Various forms of child psychopathology considered by the Diagnostic and Statistical Manual of Mental Disorders (DSM-5) have been included within this spectrum, in particular attention deficit-hyperactivity disorder (ADHD), oppositional defiant disorder (ODD), and conduct disorder (CD) [6,7].

### 1.2. Individual Differences and the Externalizing Spectrum

As noted, the externalizing spectrum includes several clinically recognized disorders such as ADHD, CD, and ODD. The core of these disorders refers to maladaptive behaviors, impulsivity, deficit in inhibitory control causing impairment, or interference, in everyday life functioning. Research efforts have focused on exploring the longitudinal association between early individual differences and adolescent/adult externalizing behaviors.

Thus, for instance, poor intellectual performance has been associated with externalizing behaviors in cross-sectional and longitudinal studies. Analyzing a sample of 2370 secondary school students, Huepe, Roca, Salas, Canales-Johnson, Riveral-Rel, Zamorano et al. [8] found that those within the lowest 5% of the intelligence distribution (assessed with the Raven’s Progressive Matrices Test) showed higher frequency of bullying behaviors and drug use. Caspi, Houts, Belsky, Harrington, Hogan, Ramrakha, Poulton and Moffitt [9] studied a cohort from the Dunedin Longitudinal Study, finding that measures of neurological functioning, intelligence, receptive language and motor skills measured at age three predict, 35 years later, problematic adult behaviors (criminality, excess weight or dependence from social welfare) (AUC = 0.87). Childhood low IQ and low self-control did also predict adult difficulties. However, Young, Friedman, Miyake, Wilcutt, Corley, Haberstick, and Hewitt [10] supported the opposite relation: measures of conduct disorder and ADHD at age 12 predicted worse executive response inhibition at age 17 (−0.41).

Personality traits such as impulsivity, aggressiveness, empathy, honesty and irresponsibility have been related to externalizing behaviors [1,11]. The link between personality traits and externalizing behaviors has been studied within the framework of the five-factor model. Based on the correlations among the five personality traits, two higher-order traits were obtained: stability (neuroticism reversed, conscientiousness, and agreeableness) and plasticity (extraversion and openness). Considering a sample of 140 adolescents, DeYoung, Peterson, Séguin and Tremblay [12] analyzed the relation between externalizing and these higher order factors. Stability was negatively correlated (*r* = −0.71) while plasticity was positively correlated (*r* = 0.75) with externalizing behaviors. Intelligence was also negatively correlated with these behaviors (*r* = −0.59) as assessed by students’ teachers.

The variance shared by stability and plasticity gives rise to a single higher order dimension: the general factor of personality [13,14]. This factor is thought to reflect social effectiveness [15]. The longitudinal association between this higher-order factor and externalizing behaviors remains unexplored, but cross-sectional findings suggest that they might be related [16]. 

Longitudinal research has focused on the predictive power of early individual differences over the onset of later (adolescence and early adulthood) externalizing behavior, whereas less attention has been devoted to the relationship between early externalizing behaviors and individual differences in intelligence and personality in adulthood. Walton, Krueger, Elkins, D’Accordo, McGue and Iacono [17] conducted a population-based longitudinal study (*N* = 1252) with participants of the Minnesota Twin Family Study who completed the Multidimensional Personality Questionnaire at age 17 and were screened for diagnoses of antisocial behavior and substance misuses at ages 17, 20, 24, and 29. Aggression scores were consistently associated with diagnoses of antisocial behavior (correlations ranging between 0.26 and 0.46), and alcohol dependence (correlations between 0.2 and 0.49). Loeber, Menting, Lynam, Moffitt, Stouthamer-Loeber, Stallings et al. [18] studied a sample of boys from the Pittsburgh Youth Study (*n* = 422). Participants completed measures of cognitive impulsivity and intelligence at age 12. Both traits predicted the age-crime curve. Criminal behavior was more frequent in boys with high cognitive impulsivity or lower IQ.

To summarize: while the relation between externalizing disorders and individual differences in several areas seems to be supported by longitudinal studies, it is unclear how the presence of these externalizing behaviors is related to adult personality and intelligence. There are several reasons to expect that early externalizing problems will have a reflection in adult individual differences. First, there is evidence that externalizing behavior presents moderate to high stability over time, at least from childhood to adolescence [19]. Second, externalizing behavior usually relates to later antisocial behavior or delinquency [20]. Third, externalizing behavior is associated with several neuropsychological deficits such as working memory and emotional self-regulation skills [21,22]. Therefore, in the present study we assume that these neuropsychological dysfunctions may have a certain degree of developmental stability and, therefore, exert a negative influence over adult personality and intellectual level.

### 1.3. Child Maltreatment

Child maltreatment might predict adult negative outcomes [23,24,25,26]. Furthermore, the experience of neglect and various forms of child abuse might be associated with future cognitive impairment [27,28], and personality profiles characterized by socialization difficulties expressed by traits such as conscientiousness, agreeableness, and openness [29,30].

### 1.4. Present Research

The following research questions will be addressed in the present research:

(1) Are individual differences in childhood externalizing behaviors associated with personality, socialization difficulties, and intelligence assessed in adulthood?
(2) Is self-reported experience of childhood maltreatment related with personality, socialization difficulties, and intelligence assessed in adulthood?

#### Personality Traits are Considered Using Two Approaches

First, the traits tapped by the five-factor model (FFM) will be analyzed for obtaining one single dimension (GFP = general factor of personality). The conceptual relevance of this general psychological construct has been exhaustively analyzed by John Loehlin [13] who concluded: “GFPs obtained as unrotated first factors have considerable generality across personality inventories and levels of measurement (…) GFPs derived from self-reports and from others’ reports were substantially correlated (…) a GFP is a fairly generalizable and quite readily measurable phenomenon” (p. 262).

Furthermore, a large-scale meta-analysis found great overlap between the GFP and emotional intelligence [31], which might support the view of GFP as a social effectiveness factor. We note, however, that Schermer and Vernon [32] found a positive, but moderate, relation between GFP and a measure of social desirability.

As highlighted by Loehlin [13], the GFP would benefit from evidence of an association with behavioral correlates. The present research looks for evidence of associations between the GFP and nearby constructs (externalizing behaviors and socialization) along with intellectual traits.

Nevertheless, we will also report associations among the traits included in the FFM—instead of the computed GFP—and the remaining variables of interest.

Second, socialization difficulties will be assessed with the socialization scale (SOC) [33] designed from Lykken’s model of antisocial personality [34]. Greater levels of sensation seeking, fearlessness, aggressiveness, and impulsivity are expected to increase vulnerability to antisocial and disruptive behaviors. Lower intelligence levels in adulthood are also expected in the presence of childhood externalizing behaviors and maltreatment episodes. Lykken’s model is based on Gray’s [35] and Zuckerman’s [36] models of personality.

In this regard, Herrero and Colom [37] administered the SOC to prison inmates (*n* = 186) and controls (*n* = 397) finding higher scores in sensation seeking and fearlessness in the former. Moreover, SOC scores haven been related to suicidal behavior. Negredo, Melis and Herrero [38] found a positive correlation between impulsiveness and the number of suicide attempts, as well as between the latter and sensation seeking.

In the present study, intellectual functioning was measured by measures of fluid and crystalized intelligence.

For analyzing the relationships among the variables of interest (childhood maltreatment episodes, childhood externalizing behaviors, adult intelligence, adult personality, and adult socialization difficulties), we considered their longitudinal associations: maltreatment episodes (retrospective self-reports) and externalizing behaviors (observer ratings) occurring in 2002, along with intelligence and personality variables assessed in early adulthood (2014–2017).

## 2. Method

### 2.1. Participants

The dataset analyzed here is based on the Longitudinal Study of Intelligence and Personality conducted by the Laboratory of Individual Differences at the Federal University of Minas Gerais, Brazil. The study began in 2002 recruiting 650 schoolchildren and they were assessed every two years until 2010 in both intellectual and personality variables. Here, we will analyze data of 120 participants (56.7% males) assessed in 2002 (T1, mean age = 10.0; SD = 2.2) who also completed a psychological assessment in 2014–2017 (T2, mean age = 23.5; SD = 2.2); 81.7% were university graduates, 11.6% had completed high school, and 6.7% had completed compulsory school.

### 2.2. Measures

As described above, externalizing behaviors were assessed in childhood (T1). These behaviors were registered using the Teacher Rating Scale for ADHD [39]. This scale measures externalizing behaviors observed in school, according to DSM-IV criteria. This scale comprises 49 items organized in four sub-scales: Attention Deficit (e.g., “he/she is easily distracted by any stimuli”); Learning Problems (e.g., “he/she is reluctant to engage in tasks that require mental effort”); Hyperactivity (e.g., “he/she always seems to be connected as an engine”); and Antisocial Behavior (e.g., “he/she is always quarreling”). According to the scale’s manual, reliability values (Cronbach’s alpha) range from 0.90 (Antisocial Behavior) to 0.97 (Attention Deficit). Two teachers provided a rating (after mutual agreement) for each child. In a previous study on a sample extracted from the same longitudinal project, Andrade and Flores-Mendoza [40] found similar reliability values. The correlation between Attention Deficit and Learning Problems was 0.90, and, therefore, for the present study we considered only three subscales (Learning Problems, Hyperactivity and Antisocial Behavior; see the remaining correlations in the Supplementary Material).

Episodes of childhood maltreatment were evaluated at T2 using the Childhood Trauma Questionnaire [41,42]. This is a retrospective self-report that includes 28 items tapping emotional, physical, and sexual abuse. According to studies published in Brazil [43], reliability values (Cronbach’s alpha) are 0.90 for emotional abuse, 0.86 for sexual abuse, and 0.69 for physical abuse. In a previous study with Brazilian males [44] similar reliability values were found.

#### Intelligence, Personality, and Socialization Difficulties Were Assessed at T2

Intelligence was assessed by eight subtests from the Wechsler Adult Intelligence Scale—WAIS-III [45]: Vocabulary, Information, Similarities, Comprehension, Digit Span, Letter-Number Sequencing, and Arithmetic. The Raven Progressive Matrices Test was also administered [46].

Personality traits were assessed by the NEO-PI-R [47]. This questionnaire taps the personality traits considered by the FFM: extraversion, agreeableness, conscientiousness, neuroticism, and openness. Reliability values (Cronbach’s alpha) were: 0.875, 0.851, 0.907, 0.914, and 0.862 respectively. Test-retest reliability coefficients (four months apart) ranged from 0.869 (Openness) to 0.943 (Conscientiousness).

Finally, socialization difficulties were assessed using the SOC scale [33]. The SOC includes three subscales: Sensation Seeking, Impulsivity, and Fearlessness. Reliability values (Cronbach’s alpha) were 0.692, 0.704, and 0.764 respectively. The reliability value for the general SOC score was 0.826.

### 2.3. Analyses

As specified at the introduction section, the interest here focuses on the analysis of the relationships between (childhood) maltreatment episodes/externalizing behaviors (T1, 2002) and (adult) intelligence/personality/socialization difficulties (T2, 2014–2017).

The descriptive statistics and correlations among all the specific measures included in the global scores are reported in the Supplementary Material.

Because of the relatively small sample size considered in the present study, we decided to analyze global scores after computing exploratory factor analyses (EFA). Therefore, five scores were obtained for computing the analyses of interest: (1) maltreatment episodes (retrospective report); (2) externalizing behaviors (Attention Deficit, Learning Problems, Hyperactivity, and Antisocial Behavior); (3) intelligence (WAIS-III and Raven matrices); (4) general factor of personality (NEO-PI-R); and (5) socialization difficulties (SOC scale).

Factor analyses (maximum likelihood) were computed for obtaining the global scores of interest: (a) the score for externalizing behaviors is based on the variance shared by Learning Problems, Hyperactivity, and Antisocial Behavior; (b) the GFP score taps the common variance of the five personality dimensions assessed by the NEO-PI-R; (c) the socialization difficulties score reflects the variance shared by Sensation Seeking, Impulsivity, and Fearlessness; (d) the Intelligence score is based on the WAIS-III subtests and the Raven Progressive Matrices.

For each latent variable, we report the factor loadings and the percentage of explained variance in the Supplementary Material. Using FACTOR 9.2 [48] we computed the factor determinacy index (FDI). When this index is close to one, the obtained factor score properly represents the latent trait of interest. Ferrando and Lorenzo-Seva [49] showed that FDI values around 0.80 are adequate for research purposes. The Supplementary Material reports FDI indices along with the estimation of the reliability of the obtained factor scores. Additional, correlations between the three used ADHD subscales and the other general constructs (socialization difficulties, intelligence and the GFP) are also reported.

The five variables of interest were organized within one path-model distinguishing those occurring or assessed at T1 (maltreatment episodes and externalizing behaviors) and those assessed at T2 (intelligence, personality, and socialization difficulties). Path models were also computed separately for the traits included in the FFM.

## 3. Results

Table 1 shows the descriptive statistics for the five variables of interest. The correlation matrix is also shown.

Table 1 reveals that: (1) maltreatment and externalizing behaviors are unrelated (*r* = 0.04); (2) maltreatment is positively related to socialization difficulties (*r* = 0.27) and negatively related to the general factor of personality (*r* = −0.40); (3) maltreatment and intelligence are unrelated (*r* = −0.05); (4) externalizing behaviors are positively related to socialization difficulties (*r* = 0.22) and negatively related to the general factor of personality (*r* = −0.28) and to intelligence (*r* = −0.24); (5) socialization difficulties are negatively related to the general factor of personality (*r* = −0.42); and (6) socialization difficulties and the general factor of personality are unrelated to intelligence.

Results shown in Table 1 indicate that only externalizing behaviors (T1) are related to the three variables assessed in early adulthood (T2). Maltreatment episodes (T1, retrospective) are related to personality factors (T2), but not to intelligence (T2). Finally, intelligence and personality fail to show any substantial relationship at T2.

Regarding the computed factor analyses for externalizing behaviors, the GFP, socialization difficulties, and intelligence, results showed appropriate FDI values [49]. Therefore, the global scores for these latent variables capture relevant shared variance (Table S2).

**Figure 1:**
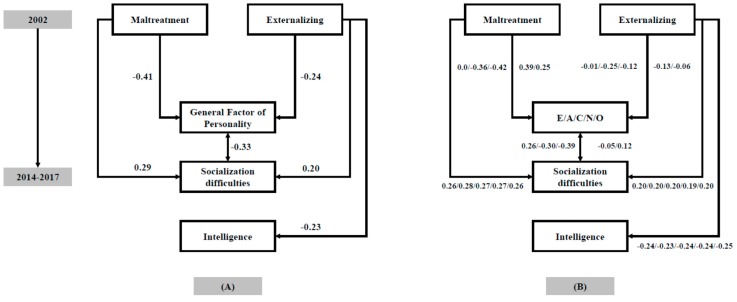
(**A**) Path analysis relating childhood maltreatment and externalizing behaviors with adult intelligence and personality variables; (**B**) Path analyses computed separately for the traits included in the five-factor model (FFM).

In the next step, the variables of interest were submitted to a path analysis considering their longitudinal nature. Results are shown in Figure 1A. Non-significant relationships are removed from the model for simplicity but reported in the text. Fit indices for this model were excellent: chi-square = 1.533 (*df* = 2, *p* = 0.465), RMSEA = 0.000, NFI = 0.97, and CFI = 1.00.

Figure 1A shows several results of interest. First, maltreatment and externalizing behaviors occurring in childhood (T1) are unrelated (β = 0.05, *p* = 0.666). Second, childhood externalizing behaviors (T1, 2002) predict the three psychological variables assessed at T2 (2014–2017): personality (β = −0.24, *p* = 0.006), socialization difficulties (β = 0.20, *p* = 0.040), and intelligence (β = −0.23, *p* = 0.019). Third, childhood maltreatment (T1, retrospective) predicts personality (β = −0.41, *p* < 0.001) and socialization difficulties (β = 0.29, *p* = 0.002), but not intelligence (β = −0.06, *p* = 0.531) (T2). Fourth, personality and socialization difficulties (T2) are related (*r* = −0.33, *p* = 0.002). Finally, intelligence is unrelated to both personality and socialization difficulties (T2). 

Figure 1B shows results when we replace the GFP with the personality traits included in the FFM. These are the main findings: (a) maltreatment is related to all traits except extraversion; (b) externalizing is related to agreeableness; and (c) socialization difficulties are related to extraversion, agreeableness, and conscientiousness. These findings suggest that all personality traits are involved with maltreatment, externalizing behaviors, and socialization difficulties, albeit the most relevant may change depending on the variable of under consideration: (1) agreeableness is relevant regarding maltreatment, externalizing, and socialization; (2) conscientiousness is relevant for maltreatment and socialization; (3) neuroticism is relevant for maltreatment; and (4) extraversion is relevant for socialization.

## 4. Discussion

Here we have considered the longitudinal associations of maltreatment episodes and externalizing behaviors occurring in childhood with psychological factors (intelligence, personality, socialization difficulties) assessed in early adulthood. The observed findings are summarized and discussed next.

First, the GFP was negatively related to socialization difficulties. Therefore, this global factor might summarize “socially effective personality traits”. Figure 1B reveals that extraversion, agreeableness, and conscientiousness may drive this relationship. Higher levels of extraversion, along with lower levels of agreeableness and conscientiousness, are related with socialization difficulties.

Second, maltreatment episodes and externalizing behaviors were unrelated. This suggests that children showing disruptive behaviors are not necessarily maltreated (and the other way around).

This finding is consistent with De Sanctis, Nomura, Newcom and Halperin [50], who observed that child maltreatment and conduct disorder were independent risk factors of adolescent antisocial behaviors.

Furthermore, McKee, Colletti, Rakow, Jones and Forehand [51] failed to find evidence for the specificity of parenting practices (warm, hostile, and control) regarding child disruptive behaviors.

According to the reported results, externalizing behaviors observed in childhood precede the configuration of complex personality traits, while self-reported maltreatment might be related to non-social personality variables. Teachers rated children’s externalizing behaviors and their assessments might differ from those provided by their parents. For instance, after studying 107 children from the same school, Andrade and Flores-Mendoza [40] reported high agreement between teachers and parents for Attention Deficit, but the agreement was lower for Hyperactivity and Antisocial Behavior.

Third, maltreatment and externalizing behaviors predicted socialization difficulties (as indexed by greater impulsivity, sensation seeking, and fearlessness) and one greater socially effective personality (as indexed by the general factor of personality, GFP) in early adulthood. These results might validate teachers’ assessments of externalizing behaviors made years before.

Maltreatment (adverse experiences) and externalizing behaviors were negatively related to the general factor of personality, whereas they were positively associated with socialization difficulties. Therefore, children showing disruptive behaviors and experiencing episodes of maltreatment do show a personality profile suggesting worse social adaptation. This finding is consistent with previous results (see [52] for a review). Interestingly, the general factor of personality was negatively related to socialization difficulties, which supports the view that the former might be a meaningful psychological dimension (rather than a simple statistical artifact) tapping social effectiveness [15].

Childhood maltreatment showed greater relationships than externalizing behaviors with the GFP. Nevertheless, we acknowledge that the reliability of self-reported childhood abuse may be a serious challenge in clinical research [53]. Here, the same individual did provide information regarding child maltreatment and personality traits, whereas teachers provided the assessment of the analyzed externalizing behaviors. This informant-dependency issue might increase the relationship in the first instance [53].

Fourth, only childhood externalizing behaviors predicted intelligence assessed in early adulthood. This is expected, given available research showing worse academic performance and lower cognitive functioning [54,55] in children and adults with disruptive behavior [56].

Surprisingly, we failed to find a significant relationship between episodes of childhood maltreatment and adult intelligence. Nevertheless, the type of maltreatment and time of occurrence may be relevant moderating variables. Thus, for instance, Nikulina and Widom [27] found relationships between maltreatment and cognitive functioning. However, only childhood neglect (but not physical and sexual abuse) predicted worse executive functioning and non-verbal reasoning performance. Jaffee and Maikovich-Fong [57] reported that chronic, but not temporary (situational) maltreatment, was related to externalizing behaviors and intelligence.

Finally, intelligence and personality were unrelated in the present dataset. Externalizing behaviors assessed in childhood predicted both psychological factors to the same extent (around *r* = 0.23), whereas self-reported episodes of childhood maltreatment predicted personality only. This suggests dissociation between adult intelligence and personality with respect to maltreatment episodes and externalizing behaviors occurring in childhood.

The interplay between intelligence and personality is highly complex [58]. Woods, Hinton, von Stumm and Bellman-Jeffreys [59] found significant correlations between a higher-order personality factor and verbal ability. However, the correlations between personality and quantitative/visuospatial abilities were non-significant. Also, null correlations between cognitive (fluid intelligence + working memory) and personality (socialization difficulties) variables were observed by Colom, Escorial, Shih, and Privado [60], although both did predict scholastic achievement to the same extent. There is also evidence from genetically informative samples indicating that *g* and GFP are unrelated at the genetic level. Loehlin, Bartels, Boomsma, Bratko, Martin, Nichols and Wright [61] studied a sample of monozygotic (*n* = 1748) and dizygotic (1329) twin pairs from different countries. The genetic correlation between GFP and *g* was zero. Further research found a positive relation between *g* and GFP. Thus, for instance, Schermen and Vernon [32] found positive correlations between GFP and a measure of *g* in two independent samples (*r* = 0.256 and 0.279, respectively).

The participants in the present study were mainly university students. However, range restriction issues can be discarded here because these students were recruited in Brazilian private universities in which there is no selection based on any academic merit.

In conclusion, here we have shown that childhood externalizing behaviors (assessed by teachers) predict (a) greater early adulthood socialization difficulties (as assessed by impulsivity, sensation seeking, and fearlessness); (b) worse social effectiveness (as indexed by the GFP) and (c) lower intelligence scores (as assessed by fluid and crystallized abilities). Lower levels of agreeableness drove the relationship between the GFP and externalizing behaviors because the remaining personality factors were unrelated to this variable (Figure 1B).

Furthermore, self-reported adverse biographical events occurring in childhood also predict greater early adulthood socialization difficulties and worse social effectiveness but are unrelated to adult intelligence. The predictive values observed for childhood externalizing behaviors and self-reported adverse biographical events were independent of each other. All the considered personality traits were related to these maltreatment episodes (except extraversion) (Figure 1B).

Finally, we acknowledge limitations in the present study. First, a lack of baseline values for personality. Second, due to attrition issues, the present report focused on variables showing small numbers of missing values in 2002. Third, the sample size in T2 was relatively small.

Despite these limitations, the reported findings support the probable utility of policies devoted to the promotion of social skills in the school setting. These policies are beginning to consider personality factors within their global framework. Here we have shown that childhood maltreatment and externalizing behaviors predict worse personality profiles and, therefore, intervention programs within the school context may help to prevent future socially ineffective and disrupting personalities.

## Figures and Tables

**Table 1:** Descriptive statistics and correlation matrix.

Variables	Maltreatment (T1)	Externalizing Behaviors (T1)	Socialization Difficulties (T2)	General factor of Personality (T2)	Intelligence (T2)
Maltreatment (T1)		0.036	0.275 **	−0.404 **	−0.053
Externalizing behaviors (T1)			0.220 *	−0.279 **	−0.239 *
Socialization difficulties (T2)				−0.420 **	0.026
General factor of personality (T2)					−0.006
*N*	97	99	111	119	120
Mean	14.4	0	0	0	0
SD	13	1	1	1	1
Skewness	1.9	1.2	0.6	−0.1	−0.1
Kurtosis	5.1	1.2	0.6	0.2	−0.4

* *p* < 0.05, ** *p* < 0.01.

## Supplementary Materials

The following are available online at http://www.mdpi.com/2079-3200/6/3/31/s1. Table S1: Descriptive statistics and correlation matrix (including all the variables considered in the study); Table S2: EFA results for each general factor considered in the study; Table S3: Correlations among the three ADHD subscales and the remaining general scores.
