# Supplementary material for: The Dissociation between Adult Intelligence and Personality with Respect to Maltreatment Episodes and Externalizing Behaviors Occurring in Childhood
Source: J Intell. 2018 Jul 9;6(3):31. doi: 10.3390/jintelligence6030031 (PMC6480752; doi:10.3390/jintelligence6030031)
Supplement: Supplementary file 1 [file jintelligence-06-00031-s001.pdf]

Journal of Intelligence

MDPI

Supplementary Material

The Dissociation between Adult Intelligence and Personality with Respect to Maltreatment Episodes and Externalizing Behaviors Occurring in Childhood

Carmen Flores-Mendoza, Sergio Escorial, Oscar Herrero and Roberto Colom

Table S1. Descriptive statistics and correlation matrix (including all the variables considered in the study).

	**1**	**2**	**3**	**4**	**5**	**6**	**7**	**8**	**9**	**10**	**11**	**12**	**13**	**14**	**15**	**16**	**17**	**18**	**19**	**20**
1		−0.007	0.207	0.049	0.089	0.238*	0.261**	0.372**	0.004	0.244*	−0.365**	−0.414**	−0.068	0.076	−0.115	−0.195	0.066	0.067	−0.222*	−0.153
2			0.461**	0.769**	0.238*	0.173	0.166	−0.093	0.067	−0.058	−0.211*	−0.076	−0.236*	−0.203*	−0.133	−0.088	−0.191*	−0.190*	−0.195*	−0.101
3				0.582**	0.188	0.111	0.140	−0.215*	−0.107	−0.075	−0.272**	−0.128	−0.184	−0.303**	−0.343**	−0.229*	−0.216*	−0.195	−0.335**	−0.239*
4					0.204*	0.161	0.136	−0.126	−0.009	−0.055	−0.237*	−0.115	−0.163	−0.192*	−0.246**	−0.081	−0.216*	−0.157	−0.233*	−0.208*
5						0.307**	0.345**	−0.081	0.250**	−0.030	−0.267**	−0.316**	−0.156	−0.065	−0.011	0.047	−0.165	0.032	−0.041	−0.138
6							0.388**	0.273**	0.173	0.130	−0.309**	−0.521**	−0.104	0.115	−0.075	−0.036	0.082	−0.021	0.027	−0.055
7								−0.088	0.116	0.207*	−0.321**	−0.244*	−0.021	0.161	0.024	0.011	0.167	0.049	−0.020	0.079
8									−0.017	0.234*	−0.076	−0.172	−0.072	0.018	−0.042	−0.053	0.001	0.019	−0.009	−0.018
9										0.244**	0.245**	0.117	−0.059	−0.021	0.025	−0.025	−0.034	−0.134	0.015	−0.151
10											0.147	0.057	0.210*	0.377**	0.150	0.118	0.373**	0.100	0.157	0.121
11												0.450**	0.068	0.043	0.061	−0.014	−0.057	−0.100	−0.047	0.021
12													0.015	−0.138	−0.047	0.006	−0.076	−0.099	−0.072	0.056
13														0.319**	0.320**	0.317**	0.371**	0.423**	0.198*	0.329**
14															0.368**	0.197*	0.572**	0.289**	0.351**	0.447**
15																0.340**	0.335**	0.313**	0.366**	0.407**
16																	0.169	0.416**	0.475**	0.460**
17																		0.324**	0.337**	0.442**
18																			0.274**	0.377**
19																				0.368**
20																				
N	97	110	99	110	111	111	111	119	119	119	119	119	120	120	120	120	120	120	120	120
Mean	14.4	28.9	36.6	14.0	6.8	5.1	4.9	50.2	51.4	49.4	50.8	48.2	11.0	12.2	13.2	11.8	13.3	11.2	12.1	51.8
SD	13	15.4	12.3	6.6	3.1	3	3.4	13.2	11.5	11.2	13.1	11.7	2.3	1.6	2.4	2.5	2.3	1.8	2.4	6.8
Skewness	1.9	1.5	0.2	1.1	−0.1	0.8	0.7	−0.4	−0.5	−0.1	0.1	−0.1	0.7	−0.4	−0.7	0.4	0.0	−0.2	0.2	−1.9
Kurtosis	5.1	0.8	−0.7	1.2	−0.5	0.1	0.0	0.1	0.5	−0.1	−0.3	0.3	1.7	0.1	0.2	0.1	−0.4	0.1	−0.1	6.2

1: Maltreatment Episodes; 2: Hyperactivity; 3: Learning Problems; 4: Antisocial Behavior; 5: Sensation Seeking; 6: Impulsivity; 7: Fearlessness; 8: Neuroticism; 9: Extraversion; 10: Openness; 11: Agreeableness; 12: Conscientiousness; 13: Vocabulary; 14: Similarities; 15: Arithmetic; 16: Digit Span; 17: Information; 18: Comprehension; 19: Letter−Number Sequencing; and 20: Raven.
* $p < 0.05$, ** $p < 0.01$.

Table S2. EFA results for each general factor considered in the study.

Externalizing Behaviors		Socialization Difficulties		General factor of Personality		Intelligence	
**Variable**	**Loading**	**Variable**	**Loading**	**Variable**	**Loading**	**Variable**	**Loading**
Hyperactivity	0.784	Sensation Seeking	0.546	Neuroticism	−0.210	Vocabulary	0.632
Learning Problems	0.623	Impulsivity	0.603	Extraversion	0.309	Similarities	0.758
Antisocial Behavior	0.973	Fearlessness	0.664	Openness	0.189	Arithmetic	0.692
				Agreeableness	0.829	Digit Span	0.649
				Conscientiousness	0.528	Information	0.716
						Comprehension	0.701
						Letter−Number Sequencing	0.661
						Raven	0.807
**% Variance**	64.97%		36.77%		22.83%		49.58%
**FDI***	0.976		0.888		0.866		0.945
**Reliability FS**	0.953		0.789		0.750		0.893

(*): FDI = factor determinacy index.

Table S3. Correlations among the three ADHD subscales and the remaining general scores.

Externalizing Behaviors (Scales)	Socialization Difficulties	General Factor of Personality	Intelligence
Hyperactivity	0.245*	−0.186	−0.256**
Learning Problems	0.188	−0.262**	−0.379**
Antisocial Behavior	0.212*	−0.223*	−0.295**

* $p < 0.05$, ** $p < 0.01$.
